# Eradicating ageism through social campaigns: An Israeli case study in the shadows of the COVID‐19 pandemic

**DOI:** 10.1111/josi.12540

**Published:** 2022-08-15

**Authors:** Sarit Okun, Liat Ayalon

**Affiliations:** ^1^ Louis and Gabi Weisfeld School of Social Work Bar Ilan University Israel

## Abstract

This study examined three social campaigns for the eradication of ageism that were undertaken in Israel during the COVID‐19 pandemic (April, 2020–May, 2021). The documentation and analysis of the campaigns were undertaken via the lens of the Theory of Change and Five Key Principles for social campaigns: planning strategically, communicating effectively, fostering community engagement, implementing key activities, and using research. We conducted desk reviews and qualitative interviews with the campaigns’ organizers. The Theory of Change implemented by the campaigns targeted self‐ageism among independent older people and/or employers of older adults. All campaigns emphasized “active aging” and “successful aging,” to decrease (self)‐ageism. The focus on one dimension of active and positive aging may result in ageism and exclusion of older people who do not fit into the category of independent and active people. The complex timing of the campaigns had influenced the degree of implementation of the five key principles. The joining of forces of different organizations and the employment of more diverse representations of old age, may facilitate the achievement of campaign goals. Finally, an empirical evaluation of social campaigns’ efforts is still necessary to gather evidence about the effectiveness of social campaigns.

## INTRODUCTION

Ageism is defined as stereotypes, prejudice, and discrimination toward people because of their age (Ayalon & Tesch‐Römer, 2018; Officer & de la Fuente‐Núñez, 2018). This multi‐faceted crisis is prevalent worldwide, occurs across institutions and societies and impacts all aspects of life (Levy et al., 2022). While ageism can refer to any age group, the bulk of the research on ageism refers to ageism directed towards older persons (Nelson, 2016). Considering the aging of the population and the detrimental impact of ageism on older adult's health and wellbeing (Ayalon & Tesch‐Römer, 2018; Bai et al., 2016; Cohn‐Schwartz et al., 2022; Levy & Macdonald, 2016; Ng et al., 2016) – it is increasingly urgent to identify ways to eliminate or reduce ageism (Levy et al., 2022; Nelson, 2016).

With the goal of changing the way we think, feel and act toward people because of their age and aging, the World Health Organization (WHO) has launched a global campaign to combat ageism in 2016 (Officer & de la Fuente‐Núñez, 2018). The campaign's report (WHO, 2021) has identified four strategies for combatting ageism. The first strategy is policy and law enforcement for age equality and legislation of new laws in the field. The second strategy is educational interventions for the decrease of ageism. The third strategy is intergenerational contact interventions: the strengthening of inter‐generational interactions (for more information on these strategies see Burnes et al., 2019; Jarrott et al., 2022; Montepare & Brown, 2022). The fourth strategy is engaging in social campaigns to combat ageism: use of media advertisement to change the negative narratives surrounding age and aging. Whereas the first three strategies are already based on evidence and have proved to be effective according to the report (WHO, 2021), there is limited research evidence concerning the effectiveness of the fourth strategy of social campaigns.

The goal of this research, therefore, was to thicken the body of knowledge by collecting, documenting, and analyzing available information about the development and execution of social media campaigns for the eradication of ageism. Similar to other social campaigns (WHO, 2021), the media campaigns presented in this paper did not focus on gathering qualitative and/or quantitative measures to test their effectiveness. Hence, data concerning the effectiveness of the campaigns were limited. Instead, we focus in this paper on describing and analyzing the social campaigns via the Theory of Change framework (Clark & Taplin, 2012) and the five key principles for social campaigns proposed by the WHO (2020).

The contribution of the present study is by providing insights and highlighting key principles essential for the planning and execution of social campaigns to combat ageism (Curryer & Cook, 2021; WHO, 2020). Following such principles hope to enhance current evidence in the field and to ensure the wise planning and use of social media to produce social change. It is our hope that lessons learnt by this study will enrich the quality of future social campaigns to eradicate ageism and will improve the reliance on research to document the effectiveness of social campaigns. Such information is highly needed to change the way we think, feel and act towards people because of their age in order to live in a world for all ages (Curryer & Cook, 2021; WHO, 2020, 2021).

### Social campaigns for the eradication of ageism during the pandemic

During the early outbreak of the COVID‐19 pandemic, the virus was presented as solely a ‘problem of older people.’ Older people were presented as ‘vulnerable,’ and ‘at risk,’ while younger people were defined as being ‘irresponsible and as people who are threatening the social order.’ Ageism during the pandemic era was evident in a variety of ways including prioritizing the layoff of older people over younger people; economic incentives tied to the age of the workers; the setting of lockdown and exit strategies based on age; as well as arguments about the ethics concerning triage and the delivery of life‐saving treatments (Ayalon et al., 2021). In other words, the pandemic has emphasized age‐biased thoughts, feelings and behaviors and intensified tension between the generations (e.g., Albarracin & Jung, 2021; Ayalon et al., 2021; Ihara et al., 2021, Meisner, 2021).

In response to the increasing presence of ageism during the COVID‐19 pandemic both in everyday life as well as in policy (Ayalon, 2020), varied social campaigns were produced in an effort to eradicate ageism around the world. For examples: The HelpAge global network had led a campaign to expose ageism (“How do we #ExposeAgeism?”), by highlighting human rights and life with dignity regardless of one's age. The EveryAGE Counts aimed to put an end to ageism in Australia by changing societal norms about older people (“We made ageism a thing!”).

### The theoretical frameworks

In this study, we used two theoretical frameworks: Theory of Change and Five key Principles – which were identified by the WHO (2020) as significant for the success of social campaigns. We examined the nature and extent of use of these theories in order to further refine the literature on the development and execution of social campaigns, especially during extreme situations as was clearly the case during the early waves of the pandemic.

The theory of Change proposes a participatory process whereby in complex social problems – groups and stakeholders articulate their long‐term goals and identify the conditions they believe have to unfold for these goals to be met (Clark & Taplin, 2012). Such a theory describes the types of interventions that bring about the desired outcomes depicted in the framework map. Rather than asking “does it work?” the goal is to work toward understanding “under what conditions does something work, and for whom?” Hence, the theory suggests a clear framework for action once desired outcomes are clearly articulated (Clark & Taplin, 2012).

In recent years, there has been an increasing use of this helpful tool for developing social campaigns (Dokhanchi et al., 2019). In the field of social campaigns, the theory of change requires that the organization explicitly states how it expects a campaign to work, and thereby to make its implicit assumptions explicit. This allows an evaluator to better understand what is being implemented and why, and to make clear connections between a given intervention and its outcomes (Anderson, 2005). Campaigns constantly reconsider and revise their theory of change as they gather data that indicate whether and how their efforts make an impact (Reinholz & Andrews, 2020).

The five key principles were proposed by the WHO (2020) for implementing future social campaigns at the local, national, and regional levels. The first principle is planning strategically: early crystallization of the expected, social theory of change, as well as participatory planning with external organizations and decision‐makers from the field. The second principle is communicating effectively – intelligent use of a variety of media outlets for presentation of verbal and visual representations of the campaign. The third principle is fostering community engagement – giving voice to a range of opinions from the community and maximum involvement with target audiences. The fourth principle is implementing key activities – using as wide a variety as possible of activities, such as, discussions, creation, workshops, art, and/or information sessions. The fifth and last principle is using research – the undertaking of studies for the monitoring and assessment before, during and after the campaign.

### The case study: Social campaigns for the eradication of ageism during the COVID‐19 pandemic in Israel

With the COVID‐19 pandemic, we have witnessed the pandemic of ageism in social media discourse and relevant policies (Ayalon, 2020; Ayalon & Cohn‐Schwartz, 2021a; Clarfield & Jotkowitz, 2020). This has raised attention to the topic of ageism and the need to act. Moreover, given that WHO (2021) call to obtain further evidence from different places around the world concerning social campaigns to eradicate ageism, we focused on the description and analysis of three campaigns for the eradication of ageism that were undertaken in Israel during the pandemic (April 2020–May 2021): “Don't fall into ageism” undertaken by Vehadarta – The Third Strength; “Active aging” of The Space for Initiatives of 60+‐; and “Age diversity” of the Ministry for Social Equality.

#### The campaign “Don't fall into ageism”–Vehadarta – The third strength

The campaign, *Don't fall into ageism*, was produced by Vehadarta – The Third Strength, a social not‐for‐profit that works to strengthen and empower the population of older adults in Israel and to bring about attitudinal change in society and the way it relates to and treats older adults. The organization was established by a group of businesspeople and media professionals who perceived the advancement of the status of this population as key to improving Israeli society. In the framework of promoting employment of older adults in Israel, they work on an initiative entitled “Experience required,” that brings together employers and interested employees who wish to continue working after retirement age. Additional information about the organization can be found on their website: https://www.vehadarta.org.il/english/.

**FIGURE 1:**
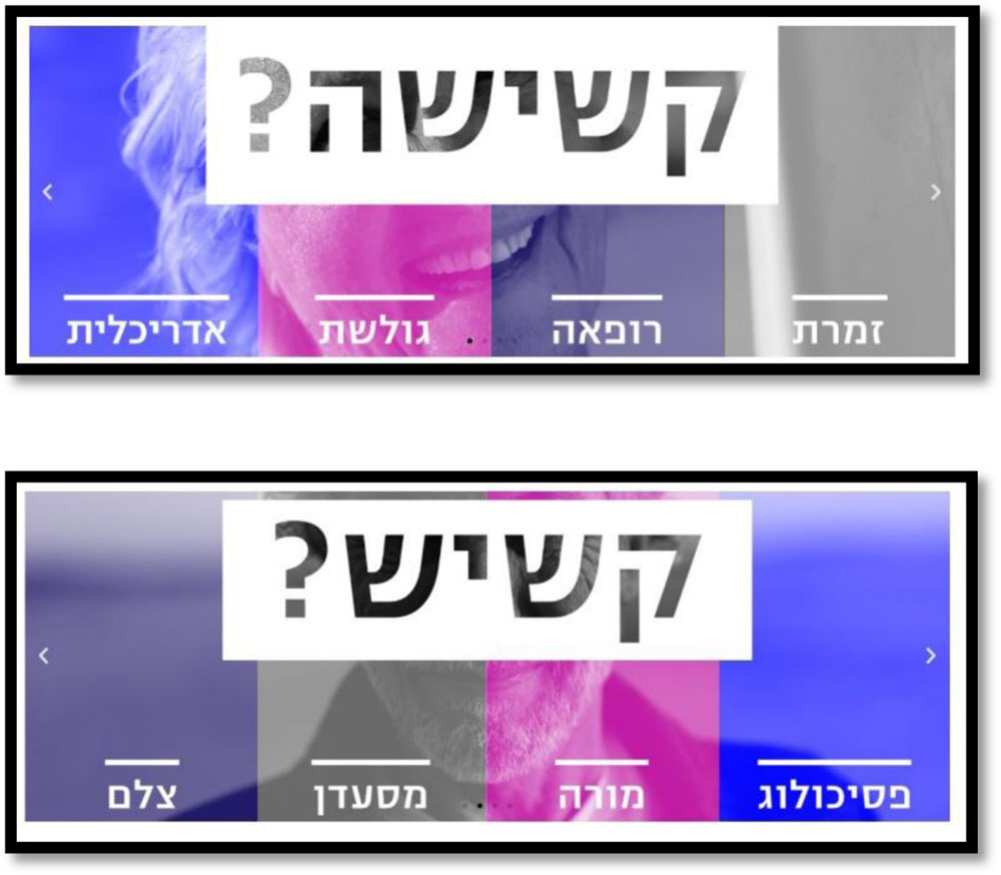
Examples of banners published during the campaign. On both banners, there are photographs of older people, whose faces are cut and blurred. In the middle of the banner there is a question, written in black: “Is this an old person?” On the bottom, there is a list of professions, such as, singer, doctor, surfer, architect, psychologist, teacher, restauranteur, and photographer. By clicking on this link – אל תיפלו לגילנות ‐ והדרת הכוח השלישי (vehadarta.org.il) (in Hebrew), you can see other examples from this campaign [Colour figure can be viewed at wileyonlinelibrary.com]

During the COVID‐19 pandemic, this organization has led a social campaign under the slogan, “Don't fall into ageism.” (see Figure 1). In this campaign, the organization aspired to engage in a joint social struggle against categorizing all older adults, 60 years or older, as an “at risk population” – a generalization that defines everyone in this group as “elderly.” This categorization led to the implementation of an economic and social closure, solely on the criterion of age. The campaign included the dissemination of protest posts by older people, the production of calls/declarations and the circulation of a petition that called for signatures. The products were published on social media, on the organization's website, and were also sent by email to a distribution list and published in the organization's newsletters.

#### The campaign “Active Aging” – The Space for Initiatives 60+‐

The campaign, *Active aging*, is the enterprise of The Space for Initiatives 60+‐ in Israel. The organization was established in partnership with Migdal (an Israeli insurance agency), a not‐for‐profit, Zionist 2000 (an Israeli association for social change), and Haifa Municipality. The Space is a HUB for social and business initiatives of senior citizens in the north of Israel and was established to encourage joint regional activities for older adults who wish to create, renew, and act for themselves and society. For more information on The Space for Initiatives 60+‐, see their website: https://www.yazamut60.org/ (in Hebrew).

**FIGURE 2:**
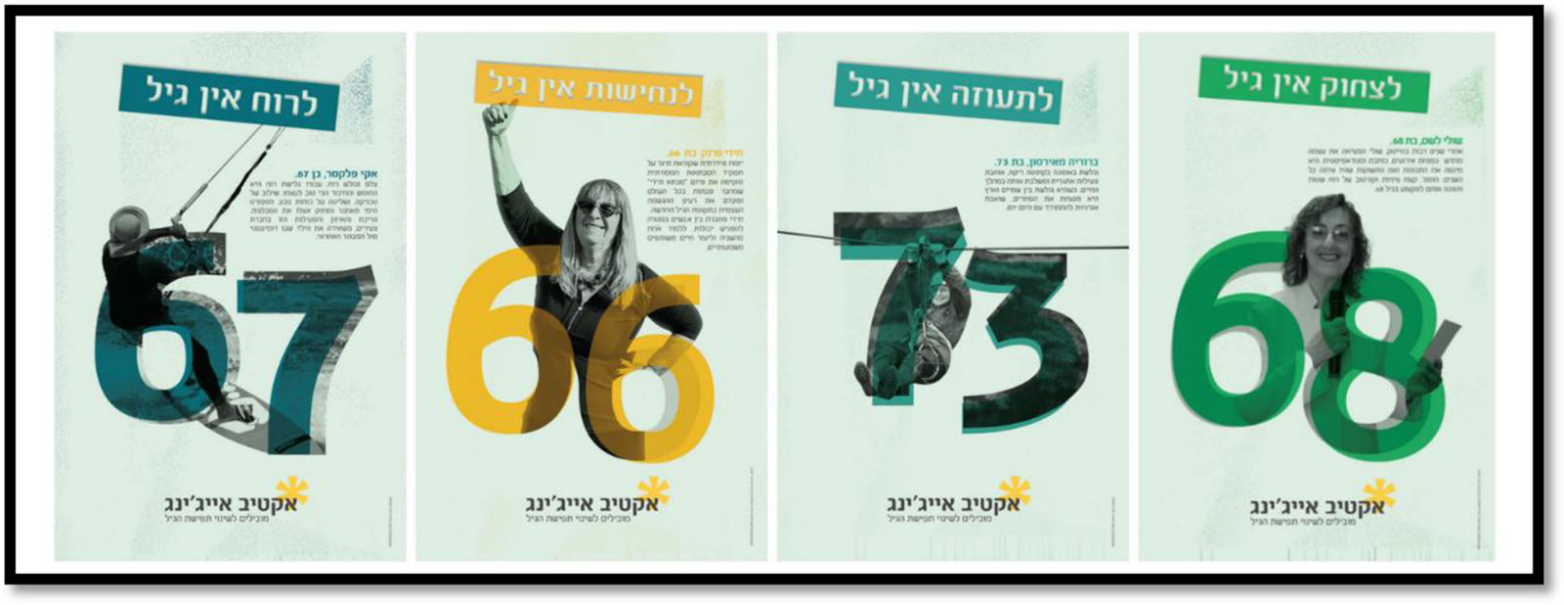
Examples of publications from the campaign. On the banner, there are photographs of members of the Space for Initiatives, showing them involved in activities that, for the most part, do not relate to older people. The person's age appears on each photo, as well as a personal story. For example, on the yellow display, there is a woman, Tiddy Frank, who is 66 years old. The text says: “Tiddy is a serial entrepreneur who challenges what it means to be a traditional grandmother.” The headline above her says: “Resoluteness has no age.” By clicking on this link: תערוכת אקטיב אייג׳ינג | yazamut (yazamut60.org), you can see a wide selection of products from the campaign's exhibition (in Hebrew) [Colour figure can be viewed at wileyonlinelibrary.com]

During the COVID‐19 pandemic, the organization organized a campaign, *Active aging*, whose goal was and remains the elimination of the ageist discourse in Israel, by changing the perception of older age in the country (see Figure 2). This campaign included six main channels of activity: podcasts, art banners/posters, personal interviews, a manifesto, a petition, and short videos. Older community members worked in the different channels, depending on their abilities, skills, and realms of interest. The social campaign's products were published and promoted on social media for 4 months. During the campaign, there were peak days, including: conferences, a festival, film launchings, and more.

#### The campaign “Age diversity” – The Ministry for Social Equality

The campaign, *Age diversity*, began as the initiative of the Israeli Ministry for Social Equality. This governmental body deals with promotion of social equality in a variety of areas and dedicates much of its time to providing rights to the senior citizens in Israel. Among its other work, the Department of Senior Citizens, in this ministry, invests in finding solutions for the problem of ageism in Israeli society. More information on this government ministry (in Hebrew) can be found by clicking on this link: https://www.gov.il/he/departments/senior‐citizens/govil‐landing‐page (in Hebrew).

**FIGURE 3:**
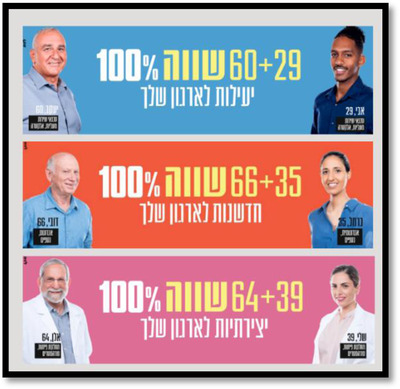
Examples of banners publicized online during the campaign. Each of these banners was disseminated on media channels and targeted different audiences. On each one, there is a pair of employees from different Israeli organizations. One represents the “older employee” and one represents the “younger employee.” Under each person, their names and ages are written, where they work and their positions in the company. For example, in the top (blue) banner, it says: 29 + 60 = 100% effectiveness for your organization. The people in the photograph are Avi, 29 years old and Ya'acov, 60 years old, both of whom work in technical support in the Elektra Company. By clicking on this link: גיוון גילי | אגף בכיר אזרחים ותיקים (www.gov.il) you can see examples of the campaign's products (in Hebrew) [Colour figure can be viewed at wileyonlinelibrary.com]

During the COVID‐19 pandemic, the Department of Senior Citizens produced a campaign for the elimination of ageism, which dealt with the importance of age diversity in workplaces in Israel (see Figure 3). This campaign was launched under the slogan: “Age Diversity – The Next Generation of the World of Business.” The campaign aspired to raise public discourse about the topic of employment of older people in Israel and presented success stories of multi‐age teams in different companies and organizations that are comprised of young and old employees. During the campaign, a website was established with content on the topic, including opinion columns, short videos, banners, and a portal for content about diversity in workplaces. Moreover, the banners and the short videos were distributed over 2 weeks via television channels and social media.

### Campaign characteristics

These campaigns were selected for several reasons. All three met the definition of the WHO concerning a “social campaign to combat ageism.” That is, they included planned activities over time to achieve an equitable and equal society for people of all ages (WHO, 2020). Secondly, the three campaigns employed the media to raise awareness concerning the phenomenon of ageism and its presence in our daily lives. Moreover, we also identified a satisfactory degree of heterogeneity; two of the campaigns were run by non‐governmental social/business organizations that work bottom‐up – whereas the third social campaign engaged in top‐down activities, because it was developed by a government ministry. Furthermore, two of the social campaigns were run by organizations that work on a nation‐wide level (the Ministry of Social Equality and Vehadarta) and the third (The Space for Initiatives) was produced by a local organization. The timing of the campaigns also varied with the Ministry of Social Equality campaign occurring almost a year after the other two campaigns.

## METHODOLOGY

The study began by undertaking a desk review of each social campaign followed by qualitative content analysis of their media products. Simultaneously, we undertook personal interviews with representatives from the organizations and analyzed them using thematic analysis.

### Data collection

The desk reviews were based on a number of informational sources: materials that were published in the media by the organizations themselves before, during and after the campaign (e.g., articles, information, pictures, posts, calls, video clips, initiatives, responses, and audio files/podcasts); materials that others reported in the media about these campaigns (e.g., news reports about the social campaigns in the local or national press); and materials that were sent to us from representatives of the organizations (such as, power point presentations and reports). In addition, we carried out personal interviews with representatives that created these social campaigns, including: the vice CEO from the Ministry for Social Equality; the head of the Community, Volunteer and Local Authorities’ Department in the Ministry for Social Equality; the head of the Employment and Housing Department in the Ministry; the CEO of the Space for Initiatives 60+; and the head of content and digital marketing for Vehadarta.

All interviews were conducted by the first author who underwent academic training in qualitative research at the Center for Qualitative Research in Israel. The authors have no personal connection to the interviewees. The interviews were conducted through telephone conversations, which were simultaneously recorded. The recordings were transcribed and analyzed thematically. The authors’ institutional ethics committee approved the study, and all interviewees provided an informed consent.

### Data analysis

After the data were collected, we undertook a qualitative content analysis of the desk reviews of each social campaign. This method is sometimes used as a basic technical instrument for the categorization of information. At other times, it is used as a tool that facilitates interpretation and the production of meaning. In any event, qualitative content analysis can provide descriptions and interpretations (Lindgren et al., 2020). In this study, we mainly used this method to describe and compare the relevant data from each campaign. In other words, the stage of data collection and categorization of the information also included the stage of first interpretation and comparison of the central aspects of each social campaign. Analysis and interpretation of the data were performed as a joint endeavor of the two researchers.

Thematic analysis of interviews. Thematic analysis is a flexible, qualitative approach to the analysis of data that can be used in a wide variety of theoretical and epistemological frameworks. This method can also be implemented with a wide variety of research questions, research designs and sample sizes (Kiger & Varpio, 2020). Even though the main goal is to capture experiences, thoughts, or behaviors (Braun & Clarke, 2006), we used it primarily to examine the ways in which the organizations’ representatives related to and gave meaning to the campaigns’ components, looking for both similarities and differences. As a result, the thematic analysis included active reading and interpretation of the interview transcripts, the search for themes that repeated themselves, and the location of unique codes in each social campaign. During the next stage, we examined if and how the different themes overlapped with the five principles identified for social campaigns. We also identified and labeled new themes. The goal of the interviews was to gain deeper understanding of the background that preceded the campaign, including its rationale and actions as well as perceptions concerning the achievements and limitations of each social campaign.

### Research team and reflexivity

The study was conducted by two researchers from the Impact Center for the Study of Ageism and Old Age at Bar‐Ilan University in Israel. The first author (PhD) is a research fellow. The second author (Prof.) is the founder and head of the Impact Center. Both authors (women) have conducted research in the field of ageism and aging and have attempted to lead a campaign to reduce ageism during the pandemic. The authors also set up a website in Hebrew and English with information on ageism to increase knowledge and awareness of the phenomenon (no2ageism.com).

## RESULTS

As can be seen in Table 1, the desk reviews provided a comparative mapping of the main components of each social campaign, including the information about the name that was chosen to describe the campaign, the role and character of the organization, the target audience, the messages, activities, media platforms and the time frame of the campaign. The following section is divided according to the Theory of Change, the Five Key Principles of social campaigns, the termination of the campaign and its products, and the timing of the campaign. This section represents an integration between the findings obtained through the desk reviews and the qualitative interviews with key representatives of each of the three organizations.

**TABLE 1:** The three Israeli social campaigns for tackling ageism during the COVID‐19 pandemic

Campaign	Organization	Vision/mission	Target audiences	Main activities	Main platforms	Time frame
Don't fall into ageism	Vehadarta	To give older people the feeling of individuality and self‐efficacy during the pandemic	1. Organization's members 2. Older people in Israel 3. General public	1. Online Banners. 2. Posts about ageism during the pandemic 3. Petition in support of the struggle	1. Email distribution lists of older people and employee 2. Social media 3.Organization's website	April–May 2020
Active aging – Leading to a change in age perception	The Space for Initiatives 60+‐	The presentation of the older age group in a new light and changing the perception of old age	1 Members of the Space 2. Older Israelis. 3. Government officials and decision makers. 4. Entire Israeli public	1. Image banners 2. Activist and protest writing 3. Establishment of a project – A wrinkle of a thought. The concept – anti‐aging 4. Short video production 5. Writing of a manifesto	1. Social media 2. The Global Virtual Museum 3. Exhibition in a Cinematheque 4. Retiree Festival 5. Online conferences 6. Space for Initiatives website.	April–August 2020
Age diversity – The next generation of the business world	The Ministry for Social Equality	Potential employers in different managerial levels, Human Resources administrators and organizational consultants	The public (including employers)	The construction of a website with content connected to the campaign, including 11 opinion pieces, three employment stories from different companies, three video testimonies from companies that have already integrated older workers, 150 different companies, a portal for content about diversity in the workplace	1. Television (mainstream channels and sector channels) 2. Digital outlets – a variety of internet sites, news sites and social media 3. The Ministry for Social Equality's website	Two weeks during May 2021
		Recruiters	Raising awareness of the organizational advantage of age diversity in workplaces			

### Theory of change of the social campaigns

We found that the Theory of Chang**e** of all three Israeli organizations was focused on the message of “active aging,” and the group of young old people. Apparently, this theory of change was chosen due to the time frame in which the three campaigns were launched. All organizations reacted to the sense of exclusion that was experienced by older people during the first wave of the COVID‐19 pandemic, which served as an engine and a motive for leading social media campaigns to eradicate ageism. During the early stages of the pandemic, older people were regarded as a homogenous at‐risk group, which should be physically isolated for the protection of older people themselves and of the healthcare systems, worldwide (Curryer & Cook, 2021). Overnight older people, some of whom highly active, were defined as an at‐risk group (Cohn‐Schwartz & Ayalon, 2021). Therefore, the desired change that the three organizations aimed at was to present older people in a positive light – as actives, productive, vital, healthy, creative, and knowledgeable members of society. As we discuss below, based on this motif, the target audiences, strategies, and creative materials were chosen

#### Don't fall into ageism

The Vehadarta organization focused on the reduction of self‐ageism of older adults by emphasizing their belonging to the period of “active aging,” and due to their being “active, independent old people.” The head of the organization's content and digital marketing explained that this focus connected to the Coronavirus:

In the first wave, all of the people who were 60+ in Israel were labeled as “old people” and as an “at‐risk population,” and it was implied by everyone that they are “non‐essential workers.” Therefore, it was important for us to give back to the older population their signs of individuality and to remind them that age is only an additional characteristic that comes after many meaningful things that they did over their lifetime… it was important for us to give older people back individuality, to cause them to hold their head up.

#### Active aging

The Space for Initiatives focused on changing the perceptions of age among and of older adults by emphasizing “active aging.” As part of the agenda for change, the organization decided to involve older adults in the process of producing the campaign. They decided not to produce the campaign for the older population, but rather to let older people produce it themselves and, in this way, to lead to perceptual changes that would begin from within. The CEO of the Space for Initiatives explained it this way:

Our goal was to create a change in consciousness among the older citizens themselves, to create in them awareness of active aging. But, it's clear that the campaign, from the first moment, targeted the entire public, society, organizations and decision‐makers. There was the clear desire here of all members of our community to respond to the exclusionary treatment that they experienced during the closures and to lead to a change on the national level.

#### Age diversityc

The government ministry focused on decreasing ageism among employers and on bringing back older people to the labor market. This campaign's goal was to lead to social change by highlighting “active aging.” Instead of creating a social campaign that emphasized equality between “young workers” and “old workers,” which according to the ministry, did not have a chance of succeeding, the ministry decided to develop a campaign that highlighted encounters between older and younger people at work and the positive consequences of such encounters. The head of the Senior Citizens’ Department explained it in this way:

We decided this time to attack ageism in the labor market by entering through the back door. The new agenda was not to tell the employer, ‘It's worth your while,’ but rather to show them why it is worthwhile to integrate older and younger workers.

### The degree of implementation of the five key principles

As can be seen in Table 2, the content analysis of the social campaigns and their products, as well as the analyses of the personal interviews, demonstrate the degree of implementation of the five key principles identified by the WHO (2021).

**TABLE 2:** The extent of implementation of the five key themes

	Planning strategically					
Campains	A theory of change	Gaining the support of decision makers	Implement‐Ing key activities	Communicating effectively	Using research	Fostering community engagement
Don't fall into ageism	Full implementation	No implementation	Partial implementation	Full implementation	No implementation	No implementation
Active aging	Full implementation	Partial implementation	Full implementation	Full implementation	Full implementation	Partial implementation
Age diversity	Full implementation	Full implementation	No implementation	Full implementation	Full implementation	No implementation

#### Planning strategically

In all three campaigns, thought was given to the planning of the strategy, including use of a Theory of Change. Nevertheless, differences were found between the organizations in relation to the issues of cooperation and contacting decision‐makers.

##### Don't fall into ageism

The producer of the campaign explained that, as a rule, the not‐for‐profit tends to work with a variety of social coalitions, engages in joint thinking, and approaches bodies that determine the tone, in terms of the social agenda. However, when the organization undertook its campaign, it did so alone because it felt the need to lead a quick and intuitive campaign during the pandemic.

##### Active aging

The heart of the planning stage of this social campaign included finding partnerships with external organizations, as well as contacting decision‐makers, so that they would help to achieve the desired change. The CEO of the Space for Initiatives explained that bureaucratic and economic support greatly influence the implementation of the social campaign and the degree of its effectiveness. She said:

There's a big advantage to being a body, which is also composed of the business sector. In my opinion, in future campaigns, there will also need to be partnerships of organizations from the first sector, the second sector and the third sector. Partnerships make flexibility and thinking outside the box possible.

##### Age diversity

The stage of strategic planning of the campaign, also included finding partnerships with external organizations and contacting decision‐makers. The representatives of the government ministry averred that this principle is part of their agenda:

We don't operate in a vacuum. We have many partners who are an integral part of the entire process, like business organizations, social organizations, government representatives and CEOs … and we have round tables, which have employers. We consult with everybody over the years, it's ongoing.

#### Communicating effectively

The three organizations dedicated resources of time and money to media aspects. The degree of implementation of this principle was reflected in the choice of the name and slogan, which provided the “first impression” of the campaign and its messages. Resources also were dedicated to choosing media channels and to their pragmatic adaptation for the target audiences.

##### Don't fall into ageism

Vehadarta chose to name its campaign “Don't fall into ageism,” and “Stop Ageism.” Use of negatives – “don't,” “fall,” and “stop” relate to ageism with negative images, such as, a trash can, a danger, a trap, and/or a phenomenon to avoid or even stop. In Hebrew, the slogan, “Don't fall into ageism,” has two forms. In the first sense – “don't fall” – cautions against sliding down the slippery slope of ageism. In the second sense – “don't fall into” cautions against committing an act of discrimination. Perhaps the double entendre of the slogan reinforced the message.

We can identify the importance of identification of the specific platforms for each target audience by noting the campaign's use of hashtags – “Stop Ageism#” and “Don't Fall into Ageism#.” The campaign's head stated that the number of tags used by the public was lower than expected and, to their regret, the spreading of the message to older surfers had failed. Their attempts to persuade older adults to add the campaign's logo to their Facebook profile photos (as often happens in the case of social campaigns) also had failed.

##### Active aging

The Space for Initiatives chose to use the name “Active Aging.” The choice of the name and its use in English rather than in Hebrew is not a given and, perhaps, decreases the understanding of some people from the target audience. While “active aging” is a catchy term, and the preferred label of the WHO (2002), by using it in Israel, the campaigners might have taken a risk of decreasing their target audience. This is probably the reason that in most of the activities that were undertaken in the framework of this campaign, the sentence “changing the perception of age” (this time in Hebrew) was added after “active aging.” The sub‐title made it possible for people who are not English speakers to connect to the message.

This campaign was undertaken solely in the digital media. All the meetings of the steering committee and of the production staff concerning the activities (video clips, podcasts, manifests, etc.) were carried out on Zoom, as well as the “peak days” of the campaign (the launch of the campaign, the virtual exhibit, conferences, and the festival).

##### Age diversity

The government ministry chose to name its campaign “age diversity.” One of the campaign's organizers explained this decision, “Today, it's very much in fashion to talk about diversity.” The idea of diversity, presented in the campaign, was the motif in all the creative materials. There were a variety of pictures, a variety of slogans, a variety of video clips, a range of ages, genders, sectors, colors, and a variety of business/employment organizations. It can be assumed that the choice of the term, diversity, aimed to harness and recruit the public for societal change that would be supportive and inclusive. The campaign was disseminated through integrated advertising, in traditional and new media, and through the targeting of different audiences.

For example, the campaign that was publicized on the website of Arabic speakers was written in Arabic so that the message would be accessible and clear to people visiting this site. On the internet sites that serve the Ultra‐Orthodox population, the ministry did not upload brochures in which there were photographs of men and women together, to be sensitive to the beliefs and Jewish laws held by this community. It is important to note that this was the only campaign that attempted to reach diverse communities.

#### Fostering community engagement

In their personal interviews, the campaigns’ organizers talked about the importance that they see in including target audiences in campaign productions. They all noted that this involvement has two goals. On the one hand, involvement raises the self‐image of the older people, while on the other, it also develops a sensitive and inclusive environment for aging and aging people. In actuality, the degree of involvement varied from campaign to campaign.

##### Don't fall into ageism

Given that this campaign aimed to eliminate ageism among and toward older people in Israel – the absence of representatives from this excluded population was conspicuous in the production staffs. The head of the campaign said that the voices of older people were provided by the organization's employees, who themselves are older adults. In other words, from their perspective, “the two hats” worn by members of the organizations’ staffs, who are themselves older adults, was perceived as involvement of older people in the production.

##### Active aging

There was wide involvement of members of the older community from the stage of planning through the stage of implementation. The campaign itself evolved from the discontent of community members concerning ageism during the COVID‐19 pandemic. They are the ones who initiated and provided the motivation for the undertaking, and they also became its main target audience.

The campaign began with a meeting of the steering committee that was comprised of representatives from the field staff of older people from the community. Older adults were recruited and worked on different functions, depending on their talents. During the stage of campaign dissemination, there was full involvement of older people. As a result, for example, in the exhibit of the products in the “Global Virtual Museum,” and in the Cinematheque's hall, the older people themselves walked around, presented and served as guides.

##### Age diversity

The involvement of the business organizations in the campaign was reflected in the participation of their representatives in the steering committee meetings and in the production. One of the organizers said, “Their presence in the meetings was significant for the process of building the campaign.” Nevertheless, community involvement only resulted in the location of companies and organizations that serve as a model for age diversity. It can be assumed that the contact made with these few companies and recruitment of employees of different ages added to the credibility of the advertising campaign. However, it remains unclear if these steps fully addressed the principle of community involvement.

#### Implementing key activities

##### Don't fall into ageism

The campaign's activity in this area was minimal and included mainly advertisement of on‐line slogans, posts on social media and the dissemination of a petition for signing. The activity amounted to making outreach to the older population and asking people to send profile photographs along with a self‐description of three to four traits, hobbies and/or profession. The idea was that their photos would be part of the advertisement‐placards. It was also further expected that there would be a significant response of older people, who would write additional posts and responses on social media in support of the campaign. However, most of the photographs that were submitted were not of a high enough quality and most of the texts were over the three to four words that the campaign organizers requested. The campaign staff had to decrease the number of photos of sufficient quality and choose a small number of words to publish.

##### Active aging

The diversity in the campaign's activities was quite noticeable and clear. This was reflected in the work of small groups, comprised of older people, who together prepared and created different initiatives for the elimination of ageism. For example, they produced video clips, recorded podcasts, and prepared art exhibits. In the framework of the activities, the older people wrote a manifesto together and sent it to government ministers and parliament members. They also disseminated a multi‐generational petition to be signed. The group of older adults who produced encouraging video clips uploaded them to social media on a daily basis. The social campaign also included the establishment of a headquarters of community members. Some members provided psychological support to Holocaust survivors and senior citizens and some bought and distributed food to older people who could not leave their homes. The organization's CEO summarized the diversity of the activities in this way, “The goal was to find different ways in which we would be able to join forces during this crisis.”

##### Age diversity

In this campaign, there were no activities undertaken with the target audiences, and it solely focused on media publication. The campaign's organizers located three to four heterogeneous employees from well‐known and respected companies. From each company, a pair of employees was selected and the two were filmed and used in campaign materials. Perhaps, for the teams of presenters, the campaign included an “activity” of a joint day of shooting. However, there was nothing beyond this.

#### Using research

All the organizations’ representatives who were interviewed for this study were aware of the importance of accompanying research for each stage of the campaign. Furthermore, they agreed that the ideal situation would be to collect data to examine and demonstrate the degree to which the change occurred.

##### Don't fall into ageism

No research was undertaken on the Vehadarta campaign, during any of the stages. According to the head of content and digital marketing, the lack of research on this campaign was due to two main reasons: budget problems (“We are a small not‐for‐profit”) and mainly because of the urgency to run a campaign due to the COVID‐19 pandemic. “This campaign was undertaken during an emergency. It was very intuitive. We didn't have time to undertake a study, because we felt that we needed to get going.” Nevertheless, she asserted that the outreach to the audience of older people and the choice of their message of raising their self‐image was influenced by academic studies concerning the harm caused by self‐ageism.

##### Active aging

While this campaign was not based on formal research of an external body, internal questionnaires and surveys were conducted during the campaign. The campaign was based on many years of professional experience of the staff. At the end of the campaign, data about exposure and involvement in publicizing materials on the social networks were collected (percentages of watching, likes, shares, etc.). Furthermore, an on‐line and off‐line questionnaire on attitudes was distributed to learn about the impact of the campaign. The organization is now awaiting its results. The CEO of the not‐for‐profit summarized it in this way: “Using the internal resources that we had, and within the limited time frame, we couldn't undertake a thorough evaluation study. However, we are definitely relating to this as a future aspiration.”

##### Age diversity

In comparison to the two other campaigns, the government ministry's entire campaign was accompanied by a wide, professional, and thorough study. The Office of Government Publications provide government ministries with the mandate to undertake both pre‐ and post‐ campaign research. In the framework of this campaign, they undertook two surveys (pre‐ and post‐), in which 508 women and men, 25 years and older, participated. The random and representative sample of Hebrew speakers was comprised of people from throughout the country. The survey examined to what extent people remembered things from the campaign as well as change of attitudes due to the campaign.

The survey results showed that 43% remembered the campaign without being prompted, 4% remembered correct details from the campaign, and 37% remembered that they were exposed to the campaign but could not remember details related to the campaign. In addition, it was found that more people remembered the digital campaign (34%) compared with the campaign on television (22%). The survey also showed that of those who were exposed to the campaign and remembered it – a third reported that they did not understand the message, and another 20% indicated incorrect details. This means that the level of comprehension of the message delivered by the campaign was limited. According to the campaign's organizers, the reason for this was a low budget and mainly the “time frame” of the campaign – although this campaign is the only one of the three that was carried out after the COVID‐19 vaccination was introduced, it was launched in Israel during a very challenging political period, accompanied by massive rocket attacks on Israel.

Regarding its effectiveness – it was found that among those who were exposed to the campaign, there was an increase in public awareness of the term “age diversity,” there was an increase in the perception of the possible contribution of age diversity to the workplace and there was an increase in the willingness of employers and recruiters to hire people over the age of 60. However, the impact was not examined over time and the study's results were not published. Moreover, the same people who developed and delivered the social campaign are the ones who evaluated it.

### The termination of the social campaigns and their results

Even though the three social campaigns officially ended, their results are still posted on the internet, on social media and on relevant websites. In other words, it appears that the endpoint of social campaigns undertaken in the digital era is unclear. Whereases the scope of investment of the organizations in promotion and distribution of the products has waned over time, “screen time” on the internet is unlimited. As a result, it is possible that the social campaigns are still having an impact (albeit, unmeasured) on internet surfers.

#### Don't fall into ageism

The results of this social campaign were not officially or quantitatively measured. However, according to the campaign’ organizer, based on an analysis of activity on social media (posts, responses, likes, shares), the impression was that the campaign was honest, sensitive, direct and left its mark on the target audience. The campaign's organizer summarized it in this way:

People felt that we are talking to them and not about them. The responses on social media included posts of identification, sharing of distress and many questions. I think that this campaign solidified the organization's activities and its status as a real address for listening and helping, not only in the field of employment.

#### Active aging

The Space for Initiatives’ campaign led to welcomed achievements on several levels. On the first, most important level, from the responses of older adults on the internal surveys and questionnaires that were disseminated toward the end of the campaign, there were strong feelings of satisfaction, mainly on the part of the older adults who had participated in the production and running of the campaign. The active participation in the campaign, and specifically the feeling of giving to others, were defined by some of the respondents as a life‐saving step. The head of the Space explained that just by being involved in the campaign's production, community members prevented feelings of frustration that older citizens had experienced in other places. “They continually told us that because they are so busy, they do not feel the surrounding limitations.”

#### Age diversity

This was the only campaign in which its organizers set the time frame to two specific weeks, because the cost of broadcasting it on television and promoting it on the internet was high. However, in this case as well, the materials and the video clips remain on the internet and are openly available. According to the organizers, in the surveys undertaken at the end of the campaign, a significant increase in public awareness was found among the public for the term, age diversity, and it was perceived by the public as admired and important. Moreover, according to the organizers, there was an increase in the willingness of employers and small businesses to hire people over the age of 60. No concrete numbers were provided, however.

### “A social campaign for an emergency situation” – The impact of the timing of a campaign

All three campaigns were planed and launched during the COVID‐19 pandemic: The campaign *Don't fall into ageism* was planned and carried out during the first wave of the pandemic (April–May 2020), when Israel went under a strict lockdown for the first time. The Campaign *Active aging* began in the first wave and continued into the second wave of the pandemic (April–August 2020). This campaign also was launched out during the first lockdown. The campaign *Age diversity* was planned during the second and third waves and carried out between the third and fourth waves of the pandemic (May 2021), after the vaccination campaign had already begun. During the 2 weeks in which the campaign was launched – there was a significant decrease in the number of infections and deaths due to COVID‐19, and many of the restrictions were lifted at that time. However, during these 2 weeks, terrorist organizations fired 4360 rockets and mortar shells from the Gaza Strip on Israel, and the security situation in Israel was unstable (Ministry of Health, 2022 – https://corona.health.gov.il/). This, according to the organizers had reduced the attention to the campaign. In other words, social campaigns do not take place in a vacuum. They highly influence and are influenced by political and social events.

#### Don't fall into ageism

The organizer of the campaign from Vehadarta explained that the campaign itself was due to the strong feelings of oppression of the older population during the first few months of the pandemic.

This was an emergency campaign. During the first wave, in the country, there was an atmosphere of oppression. There was unequivocal ageism. The everyday language that was used became very generalized and full of ageism. The thing that we found to be the most noticeable was that this stigma was only directed at old people. No other group in the population received such labels and generalizations. There was erasure of any sign of individualism; it erased the identity. In 1 day, they abolished people's pasts, all their experiences, all their traits and hobbies and definitions. People related to older adults with coarse generalization, only via their age.

#### Active aging

The CEO of the Space for Initiatives explained this in an unequivocal manner:

The campaign is without a doubt a direct result of the Corona. The Corona period was very dramatic for everyone, but especially for everything that concerns independent senior citizens… in one moment, the country decided that anyone over the age of 60 needs to be isolated. They related to all the senior citizens as one, without any differentiation. It was clear to us that there's a need to act to correct this…

#### Age diversity

The government ministry had organized campaigns in the past that promoted employment of senior citizens. According to the vice CEO of the Ministry for Social Equality, after the third vaccination in Israel, a good opportunity to launch the campaign was identified.

After the vaccinations, the difficulty of the senior citizens was derived from the cessation of the pensions and the grants given to whoever was sent to unpaid vacation or whoever was ousted from the labor market; so, the safety net of many of them decreased. At the same time, we identified that in the labor market there was a large shortage of workers. This wasn't a campaign that was solely relevant for the pandemic because it was derived from the worldview that opposes ageism, in general, but during the pandemic, its importance became clearer.

However, during the 2 weeks of the campaign, thousands of missiles were fired at Israel. This means that even though the campaign was perceived as important in the eyes of the Israeli public, people were preoccupied with life and death issues rather than with the messages advocated by the social campaign.

## DISCUSSION

Every second person in the world holds an ageist attitude toward her/himself or toward others (Officer & de la Fuente‐Núñez, 2021). Since the onset of the COVID‐19 pandemic, we have witnessed a surge in hostile ageism (e.g., Albarracin & Jung, 2021; Ayalon et al., 2021; Ihara et al., 2021), alongside numerous instances of benevolent ageism, which is sometimes an insidious form of prejudice (Ng et al., 2022). The heart of the problem is that ageism leads to a decrease in mental (Bai et al., 2016), physical health (Cohn‐Schwartz et al., 2022; Ng et al., 2016), and even to early death (Levy et al., 2002). As a result, today, perhaps more than ever, there is a need to work on local and global levels to combat this serious phenomenon (Levy et al., 2022; Nelson, 2016).

One strategy for eradicating ageism, which does not have enough empirical support is social media campaigns (Officer & de la Fuente‐Núñez, 2018). This strategy is based on the perception that the media has the power and force to help bring about behavioral change in mass populations (Meisner, 2021; Wakefield et al., 2010). Even though our study does not provide empirical evidence for the effectiveness of this strategy, this paper contributes by highlighting important insights from three different campaigns conducted within an extreme period in human history.

We found that the three organizations that led campaigns for the eradication of ageism in Israel are aware of the importance of planning strategically and of the use of online/offline media. Therefore, they dedicated resources of time, money and thought to these two principles. However, they failed to incorporate a strong evaluative component in their campaigns. Such an evaluation should have included both formative and outcome perspectives. The COVID‐19 pandemic and the ageism pandemic which followed required a quick and urgent response in the form of social campaigns that tended to neglect the evaluative aspects of the campaign.

In the analysis of the Theory of Change of each campaign, we found that they all focused on eradication of ageism among and for “active older people.” In accordance with this, they avoided engaging in activities for the elimination of ageism among and for older populations with disability and impairments that often are termed, the fourth age (Gilleard & Higgs, 2010).

The focus on this specific group of older people was expressed in each of the campaigns – each used ideas, visual and verbal materials that reflected the stereotypes of “active and contributing aging.” This motif was reflected in the content, the choice of target audiences and, most importantly, in appearances and the homogeneous age and characteristics of the people in the photographs (based on their appearance, the campaigns presented mainly people between the ages of 60 and 70 were photographed). Although the use of this specific motif was defined as part of the theory of change of the three campaigns, this finding is problematic because the sole focus on eliminating ageism among the population of “independent older people” reinforces the exclusion of older people who do not meet the “successful aging” criteria. It is interesting that the representation that was chosen is very similar to the age representations of “healthy and young” older people by dating English‐speaking websites (Gewirtz‐Meydan & Ayalon, 2018). In other words, the messages are the same – aging is a direct continuation of middle age.

Similarly, the analysis of the Theory of Change of each campaign demonstrated that they preferred messages connected to the labor market (such as, being active in developing business initiatives/age diversity in the labor market/emphasizing the profession in self‐definition). Age discrimination in the workplace including discriminatory practices based on age have deleterious effects on older workers and thus, should be eradicated (Macdonald & Levy, 2016; North & Fiske, 2016). However, the problem of mainly focusing on employment is that ageism exists in all facets of life (Ayalon & Tesch‐Römer, 2018). Moreover, even if we are to focus on the eradication of ageism in only one area, research has shown that older people are more exposed to ageism by service providers and that ageism in the labor market is turned more towards young adults (Chasteen et al., 2021). Focusing on the labor market, furthermore, reflects a neo‐liberal approach that emphasizes the contribution of older people to the economy and ignores other aspects of aging (Allen et al., 2021).

The two conclusions described above indicate that the Theory of Change that was demonstrated in these Israeli case studies reinforces the stereotype of ‘successful aging’ (Rowe & Kahn, 1997). This stereotype has been presented in previous research on ‘visual ageism’ from a global perspective (for example, Ivan et al., 2020; Kenalemang, 2021; Loos & Ivan, 2018), which showed that most of the media representations describe older adults as happy, healthy, good looking, and young, who lead dynamic lives. This partial display of the older population recycles attitudes of ageism and forces people to construct themselves as ageless individuals (Ayalon, 2021). This means that campaigns’ organizers need to take care to neither glorify nor to exclude different groups of older people and to allow for truly diverse representations of age and aging.

In addition, the analysis found that the organizations used the term, ageism, either in the campaign's name or in the campaign's slogan. Some of them propose fight the phenomenon without explaining the term at all. The issue of using the term, ageism, is indeed complicated. On the one hand, the use of the term can help disseminate the term in society and, as a result, raise awareness about this social phenomenon (Okun & Ayalon, 2022). On the other hand, in Israel and possibly in many other countries, there are people who are unfamiliar with this concept either in the local language or in English (Okun & Ayalon, 2022). As a result, it is possible that some of the public will not understand at all what the campaign is focused on. Thus, there is a real fear of losing “the audience.” Perhaps the solution is to teach the public the concept before using it directly in a social campaign.

The three social campaigns have made extensive use of digital media. Similar to a brief online intervention to reduce ageism reported in this volume (Lytle & Levy, 2022), we also found that the use of digital channels in the campaigns can be of great assistance in fostering community engagement and implementing key activities, as digital activism almost ubiquitously involves bringing together conflicting groups who are active and social media‐savvy (Vardeman & Sebesta, 2020). Therefore, the involvement and the activities did not end with participation in the filming of each campaign, but rather in the motivation for action: the writing of posts, responses, tags, likes, online registration, etc. This finding is very important for the fight against ageism because digital channels can provide more voices of silenced and excluded people. In this way, it can contribute to the strengthening and empowerment of social justice (Wakefield et al., 2010).

Clearly, all organizations knew that “older persons” represent a heterogeneous group (Gutterman, 2022) at least in terms of gender and race. This acknowledgement of heterogeneity is mainly reflected in the fact that the visual illustrations included women and men, and some of the messages were adapted to an Arabic‐speaking audience and an ultra‐Orthodox audience. However, only the campaign produced by the Ministry of Social Equality, used online as well as traditional media. Running a campaign that is entirely online is selective, because the percentage of people, 65 or older, who use the internet is low, in comparison to the rest of the population (In Israel, for example, 82% of those between the ages of 65 and 74 but only 56% of those 75 and older use the internet (Central Bureau of Statistics, 2021). As older people were the target population of all three campaigns, it is possible that some portions of the population were completely ignorant to the social campaigns. This means that on the one hand digital campaigns to eradicate ageism can contribute to strengthening social justice at a relatively low cost (Wakefield et al., 2010), and on the other hand, the “digital divide” can exclude certain target audiences. This finding is important for the fight against ageism via campaigns because it emphasizes certain susceptibilities of the target population, namely older people with limited access to the social media (Gutterman, 2022). Moreover, this attempt to target diverse populations is important given cross‐cultural differences in the experiences of ageism found in Israel (Ayalon & Cohn‐Schwartz, 2021b), and in other countries around the world (Vauclair et al., 2017). As already noted, the campaigns had failed to address diversity in old age, diverse functional ability, and different levels of involvement in the workforce and in society at large. Thus, to the most part, the campaigns targeted and represented a homogenous group of successful agers, while ignoring intersectionality.

Even though the organizations acknowledged the importance of using research, to the most part, ongoing research was missing from the campaigns’ processes. It turned out that even when there was research (like in Age Diversity, and partially in Active Aging), the results were not published and not available to the public. This possibly highlights the rapid and urgent manners in which the campaigns were constructed. The lack of research and/or publications is problematic because there is the risk that public funds and efforts will be wasted on strategies that do not achieve their goals and may even be harmful for society (Lefebvre et al., 2020).

It is recommended that social campaigns to eradicate ageism which take place at times of emergency, as was the case during the current pandemic, adopt the concept of *action research*, which involves a spiral of steps, each of which is composed of a circle of planning, action and fact‐finding related to the result of the particular action (Lewin, 1946). According to Dege (2019), there are advantages to using cycles of action because each subsequent perception steers the next action.

Finally, the COVID‐19 pandemic that reinforced the ageism pandemic in Israeli society as well as globally has served as a significant catalyst for Israeli organizations to run social media campaigns to combat ageism. These social procedures opened an important window of opportunity to document and analyze evidence concerning the use of social media campaigns as a strategy to combat ageism. Despite the variance and uniqueness of the characteristics of each organizational campaign, all three worked for the identical goal of change attitudes, stereotypes and behaviors that are age biased and to change laws and institutions that perpetuate ageism. Taking into consideration that each organization operated on its own for the same goal, in a small country, such as Israel, perhaps it would be better if the organizations were to pool their resources and work together on this national task of elimination of ageism. This idea of pooling resources between the public and private sectors for the good of a social struggle was briefly noted by Officer and de la Fuente‐Núñez (2018) and gained validation in the present study.

Social policy‐wise, our findings highlight several principles: First, the importance of acknowledging diversity related to old age and aging, as “older persons” represent a heterogeneous group spanning a range of chronological ages, functional levels, and socio‐economic contexts. Second, the need for evaluation research to accompany social campaigns, even if conducted during stressful times. We suggest the possibility of adopting action research to mitigate some of the challenges associated with this requirement. Third, the possible benefits associated with combining efforts of different organizations, rather than work carried out separately by the different organizations should be highlighted. The advantages and disadvantages associated with the use of digital media also are evident.

Nonetheless, the study has some limitations that should be acknowledged. During the first wave of the pandemic, we – the authors – began meeting with different Israeli organizations and activists to create a social campaign to combat ageism. After several meetings of the steering committee and the construction of materials, our joint work dissolved. We will not detail here the reasons why our planned campaign did not materialize, but it is important to note this information, because the analyses of the campaigns in our study were influenced by our personal experiences. The lack of data concerning planned, evaluation research of the three campaigns also limits our ability to reach conclusions about the effectiveness of the social campaigns. Evaluation research that concerns both formative aspects of social campaigns as well as their short‐ and longer‐term outcomes is highly needed to support the evidence behind social campaigns.

Moreover, it must be remembered that this study was carried out during the fifth wave of pandemic in Israel, and the threat of the virus has not yet ended. As a result, we recommend expanding and deepening the body of knowledge that has been accumulated by examining other social campaigns. We believe that by collecting additional evidence and data, we will be able to realize the vision of “a world for all ages.”

## CONFLICT OF INTEREST

The authors do not have any conflicts of interest to report.

## INSTITUTIONAL REVIEW BOARD (IRB)

Before we commenced data collection, the institutional review board at the author's university examined and approved the study.
